# FANCD2 as a ferroptosis‐related target for recurrent implantation failure by integrated bioinformatics and Mendelian randomization analysis

**DOI:** 10.1111/jcmm.70119

**Published:** 2024-10-14

**Authors:** Yuanyuan Zhou, Yujia Luo, Wenshan Zeng, Luna Mao, Fang Le, Hangying Lou, Liya Wang, Yuchan Mao, Zhou Jiang, Fan Jin

**Affiliations:** ^1^ Department of Reproductive Endocrinology, Women's Hospital, School of Medicine Zhejiang University Hangzhou China; ^2^ Department of NICU, Sir Run Run Shaw Hospital, School of Medicine Zhejiang University Hangzhou China

**Keywords:** ferroptosis, immune infiltration, machine learning, Mendelian randomization, recurrent implantation failure, single cell

## Abstract

Despite advancements in assisted reproductive technology, recurrent implantation failure (RIF) remains a challenge. Endometrial factors, including ferroptosis and immunity, may contribute to this issue. This study integrated bioinformatics analysis and Mendelian randomization (MR) to investigate the expression and significance of DEFRGs in RIF. We intersected 484 ferroptosis‐associated genes with 515 differentially expressed genes (DEGs) to identify key DEFRGs. Subsequent analyses included enrichment analysis, molecular subtype identification, machine learning model development for biomarker discovery, immune cell infiltration assessment, single‐cell RNA sequencing, and MR to explore the causal relationships of selected genes with RIF. In this study, we identified 11 differentially expressed ferroptosis‐related genes (DEFRGs) between RIF and healthy individuals. Cluster analysis revealed two distinct molecular subtypes with different immune profiles and DEFRG expressions. Machine learning models highlighted MUC1, GJA1 and FANCD2 as potential diagnostic biomarkers, with high accuracy in RIF prediction. Single‐cell analysis further revealed the cellular localization and interactions of DEFRGs. MR suggested a protective effect of FANCD2 against RIF. Validation in RIF patients confirmed the differential expression of key DEFRGs, consistent with bioinformatics findings. This comprehensive study emphasize the significant role of DEFRGs in the pathogenesis of RIF, suggesting that modulating these genes could offer new avenues for treatment. The FANCD2 is a potential gene contributing to RIF pathogenesis through a non‐classical ferroptosis‐dependent pathway, providing a foundation for personalized therapeutic strategies in RIF management.

## 1 INTRODUCTION

In the past few years, the most successful approach for treating infertility has been assisted reproductive technology, specifically in‐vitro fertilization and embryo transfer (IVF‐ET).^1^ The success of IVF‐ET relies heavily on embryo implantation, which is the initial stage of human reproduction. Recurrent implantation failure (RIF) is a clinical occurrence where multiple embryos fail to attach after transfer, impacting approximately 10% of couples who undergo IVF‐ET.^2^ In the present clinical setting, RIF is typically characterized as the inability to achieve a clinical pregnancy following the transfer of a minimum of four high‐quality embryos in a minimum of three cycles, whether fresh or frozen.^3^ Although there is an increasing amount of literature regarding RIF, a universally acknowledged definition or standard procedure for diagnosing and treating RIF is yet to be established.^4^ As a result, the prevention and prediction of RIF are hampered, leading to an increased risk of overdiagnosis and treatment, with significant physical, mental, and socio‐economic consequences for women.

Treatment of RIF is challenging due to insufficient understanding of its pathogenesis. Previous research has indicated that the emergence of RIF could be influenced by a range of factors including immunological,^5^ genetic,^6^ haematological,^7^ anatomical irregularities,^8^ microbiome^9^ and endocrine elements.^10^ Among them, abnormal endometrial function is a key factor in RIF as it provides the environment for the implantation of the developing embryo. Franasiak and Ma et al. reviewed the endometrial‐related factors in the development of RIF, including endometriosis, adenomyosis and chronic endometritis.^4^, ^8^ Approximately two‐thirds of all implantation failures were attributed to impaired endometrial function.^11^ Through single‐cell transcriptome analysis, Lai et al. found that compared with controls, there were significant differences in endometrial permissive gene expression in endometrial fibroblasts from patients with RIF, and the endometrial microenvironment was disturbed.^12^ The precise mechanism is still uncertain, but endometrial dysfunction significantly contributes to the development of RIF.

Ferroptosis, an emerging focus in the realm of female reproductive disorders, is a unique type of programmed cell demise distinguished by iron overload and lipid peroxidation, setting it apart from autophagy, apoptosis, and necrosis.^13^, ^14^ Excess or insufficient ferroptosis is associated with many physiological and pathophysiological processes, as well as with dysregulated immune responses.^15^ According to recent research, there is a significant correlation between ferroptosis and infertility as well as miscarriage caused by endometrial factors. Nong et al. found that disruption of endometrial function and signalling pathways, caused by changes in the expression of iron‐death‐associated genes, can result in recurrent pregnancy loss and unexplained infertility (UI).^16^ Hu et al. indicated that elevated ferroptosis in the uterus and placenta is linked to foetal miscarriage caused by oxidative stress.^17^ However, the relationship between ferroptosis and RIF remains unclear.

The utilization of gene microarray sequencing technology has provided numerous researchers with the opportunity to share resources and experience enhanced convenience.^18^ In addition, Mendelian randomization (MR) has been widely used in genetic epidemiological research design in recent years, which uses genetic variation as a natural experiment to improve causal inferences drawn from observational data.^19^ Nevertheless, thus far, there has been no implementation of a holistic bioinformatics strategy and MR analysis to investigate the mechanism of genes associated with ferroptosis in RIF. Therefore, we explored the pathogenesis of ferroptosis in RIF from a multidimensional perspective using genetic data, transcriptomic data, and single‐cell data to provide new insights into the diagnosis and treatment of RIF in the clinic.

## 2 MATERIALS AND METHODS

### 2.1 Data source and pre‐processing

In the research, we acquired a microarray of gene expression from the GEO database (http://www.ncbi.nlm.nih.gov/geo). Endometrial tissue samples in GSE111974 dataset were collected from 24 patients with RIF and 24 fertile control patients at Istanbul University School of Medicine during 2014–2015 for a prospective cohort study.^20^ Additionally, GSE183837 is a dataset consisting of endometrial single cells from six RIF patients and three fertile control patients.^12^ The FerrDb database, (http://www.zhounan.org/ferrdb/index.html [accessed on 11 November 2023]), represents the first validated repository of ferroptosis function‐related data. Within this database, a total of 484 ferroptosis‐related genes were identified, with 11 designated as ferroptosis markers, 264 as ferroptosis drivers, and 238 as ferroptosis inhibitors. Additionally, 29 genes were found to be annotated in multiple functional groups.^21^

### 2.2 Identification of differentially expressed genes (DEGs) and functional enrichment analyses

The GSE111974 data were normalized and identificated for DEGs with ‘limma’ package. DEGs were identified by establishing a threshold with |log_2_FC| > 1 and *p* < 0.05. Subsequently, the differentially expressed ferroptosis‐related genes (DEFRGs) were acquired for subsequent examination. The chromosomal positions of significant DEFRGs were mapped using R package ‘RCircos’, and correlation coefficients were calculated using Spearman correlation. In order to investigate the functioning of DEFRGs in RIF, the R package ‘clusterProfiler’ was employed to conduct Gene Ontology (GO) and Kyoto Encyclopedia of Genes and Genomes (KEGG) enrichment analyses.

### 2.3 Construction and evaluation of machine learning models

To diagnose RIF, machine learning models were utilized to acquire optimal DEFRGs.^22^ To begin with, the 11 genes shared between diseases were inputted into the LASSO algorithm, which can effectively handle high‐dimensional data by performing variable selection and regularization, enabling it to retain the most important predictors and shrink the coefficients of less important variables to zero. Utilizing the ‘glmnet’ package with cross‐validation of tenfold, we developed a regression model. With the ‘family’ parameter set to “binomial,” the best lambda value was chosen by ‘lambda.min’. SVM‐RFE is a feature selection method based on a linear SVM weight vector that recursively removes the least important features, which helps to identify the most relevant feature genes for dimensional genomic data. Cross‐validation ten‐fold identified the variable with the lowest classification error by ‘e1071’ package. RandomForest is an ensemble learning method that combines multiple decision trees to improve classification accuracy and robustness. This helps reduce the risk of overfitting and ensures that our model generalizes well to new data, which is critical for reliable biomarker discovery. In order to determine the optimal number of trees in the discovery cohort, the randomForest method constructed a random forest model based on 500 trees from the discovery cohorts by ‘randomforest’ package. After ranking genes by importance, we plotted the 11 significant genes in disease group for each Gini threshold of 2. Finally, we used ‘VennDiagram’ package to intersect the optimal genes of the above three algorithms. To showcase the diagnostic capabilities of the primary DEFRGs, a receiver operating characteristic (ROC) curve was performed and the area under curve (AUC) was evaluated for efficiency and accuracy by ‘pROC’ package.^23^

### 2.4 Cells immune infiltration analyses

To score immune cells enriched in samples, the single sample gene set enrichment analysis (ssGSEA) approach from ‘gsva’ package was employed. The outcomes were displayed through heat maps and violin plots created using the ‘corrplot’ and ‘ggplot2’ packages in R. The correlations between immune infiltrating cells and significant genes were evaluated by the R statistical package through the utilization of Spearman correlation coefficients.

### 2.5 Identification of molecular subtypes based on DEFRGs

The process of consensus clustering involved multiple iterations of the K‐means algorithm to obtain the input partition. Subsequently, the common matrix was calculated using the resulting partition. The primary objective was to identify disease subtypes that exhibit comparable traits.^24^ To generate subtypes with distinct features, the R software's ‘ConsensusClusterPlus’ package was employed for clustering RIF samples that had significant DEFRGs.^25^ The optimal number of clusters was determined after assessing the consistency scores, consistency heat map, cumulative distribution function (CDF) and CDF delta area curve. The optimal grouping result is determined by finding the value of *K* that corresponds to the approximate maximum value of CDF.^26^ Set the maximum value of the *K* value representing the number of clusters to 9. To assess if the classifications of RIF patients accurately represented their characteristics, the classified samples underwent principal component analysis (PCA).^27^ Moreover, the levels of expression of important DEFRGs were additionally examined to ascertain if there were significant differences between molecular subtypes.

### 2.6 Processing of ScRNA‐seq data and identifying cell types

Single‐cell RNA sequencing was employed to analyse the cellular localization and interactions of DEFRGs in the endometrium. This technique allowed us to profile gene expression at the individual cell level, revealing specific cell types and their interactions. The detailed workflow includes cell isolation, RNA extraction, sequencing, and subsequent bioinformatics analysis to interpret the data. Raw data in the form of fastq files were obtained from the GSA database and processed using Cellranger from 10X genomics to generate unique molecular identifier matrices with default settings. The R package ‘Seurat’ was used to analyse the processed data for single‐cell genomics analysis. To conduct further analyses, we removed cells that had fewer than 200 expressed genes and mitochondrial gene counts that exceeded 40%.^12^ Afterwards, the expression matrix underwent standardization by utilizing the log_2_ (CPM + 1) values as the input matrix. PCA was conducted after identifying high variable genes by applying the ‘FindVariableGenes’ function. Uniform manifold approximation and projection (UMAP) was the technique used for reducing dimensions in single‐cell visualization. Next, the ‘FindAllMarkers’ function was utilized to conduct differential expression analysis, considering |log_2_FC| > 0.3 and p.adjust <0.05. The single‐cell figure was exhibited using the ‘DimPlot’ features, while the gene expression figure was exhibited utilizing the ‘FeaturePlot’ function. Afterward, the subcategories of individual cells were labelled using the ‘HumanPrimaryCellAtlasData’ feature in the ‘celldex’ software. The calculation of cellular crosstalk was performed using the ‘CellChat’ package.

### 2.7 MR analysis

MR is a method of causal inference that leverages genetic variation to estimate the effects of biological factors on diseases by exploiting the random assortment of genotypes and their corresponding phenotypic outcomes in nature. In this study, the ‘TwoSampleMR’ package in R was utilized to extract single nucleotide polymorphism (SNP) data associated with diagnostic genes as exposure variables from the GWAS database (https://gwas.mrcieu.ac.uk/). As RIF is a specific type of miscarriage, these data were then analysed in relation to the outcome variable of spontaneous abortion from FinnGen consortium. The source information of GWAS data was presented in Table S1. To identify SNPs significantly associated with exposure factors, we incorporated all SNPs meeting the genome‐wide significance threshold (*p* < 5 × 10^−6^) while also applying stringent criteria to prune SNPs with pairwise linkage disequilibrium (LD) values <0.1, and clumping distances >100 kb. The IVW method, considered the primary method for assessing causality, yet the MR‐Egger method and weighted median method were used as auxiliary methods, yielded a nominally significantly correlated result when the p < 0.05. Additionally, The MR‐Egger intercept was used to test pleiotropy, and Cochran's *Q* test was used to estimate the heterogeneity of the SNPs. A significance level of *p* < 0.05 was deemed to be statistically significant in the assessment for heterogeneity and pleiotropy. Leave‐one‐out analysis was also conducted to identify SNPs with potential extreme influence on estimates and further evaluate the reliability of the results.

### 2.8 Human specimen immunohistochemistry and qRT‐PCR assay

This study was approved by the Ethics Committee of the Obstetrics and Gynaecology Hospital of Zhejiang University School of Medicine (Approval No. IRB‐20230391‐R). Fresh endometrial specimens from 5 RIF and 5 controls (with at least one live birth and no infertility) were collected from patients who underwent hysteroscopic surgery in the Reproductive Endocrinology Department of this hospital. In addition, 5 RIF and 5 control wax‐embedded endometrial specimens were obtained from the pathology department of this hospital. All patients were >18 years old and gave written informed consent. Sections of paraffin‐embedded specimens were incubated at 65°C for 20 min and dewaxed with xylene and alcohol before staining experiments. Following the inhibition of natural peroxidase activity using 3% H_2_O_2_ for a duration of 10 min, the antigen was subsequently retrieved in a sodium citrate buffer (pH 6.0) under elevated pressure at 120°C for an extra 15 min. After blocking with Bovine Serum Albumin (A8020, Solarbio, China), sections were incubated with primary antibodies overnight at 4°C. Nonspecific rabbit IgG was used as a negative control. On Day 2, sections were incubated with goat anti‐rabbit secondary antibody (RCA054, Shanghai Ruchuang Biological Technology CO., LTD, China), stained using a 3,3′‐diaminobenzidine peroxidase substrate kit (RCD002, Shanghai Ruchuang Biological Technology CO., LTD, China), and counterstained with haematoxylin. The primary antibodies used in the study are as follows: anti‐GPX4 (1:500; #R24461; Zenbio, China), anti‐MUC1 (1:500; #R25037; Zenbio, China), anti‐GJA1 (1:500; #340279; Zenbio, China), anti‐FANCD2 (1:500; #R381321; Zenbio, China). Five 20× magnification regions were analysed per section. The ImageJ (version 2.0.0) toolbox plugin was used to identify immunoreactive areas. Immunoreactivity is expressed as percent area (immunoreactive positive area/total area of region of interest). TRIzol‐chloroform was used to extract total mRNA from tissues (Invitrogen, USA) for qRT‐PCR. The cDNA was transcribed in reverse using a SYBR Prime Script RT‐PCR kit (#RR047, TaKaRa, Japan). The ViiATM 7500 Real‐Time PCR system was utilized for conducting PCR analysis. In the end, the fold change was determined by calculating 2 raised to the power of −ΔΔCt (2 −ΔΔCt). Table S2 contains a summary of the qRT‐PCR primers.

### 2.9 Statistical analysis

Statistical computations were performed using the R software (version 4.0.2). To compare discrepancies among the groups, the Wilcoxon rank‐sum test was employed. Correlation coefficients were calculated using Spearman correlation analysis, including between DEFRGs, as well as between DEFRGs and immune cells. In order to determine statistical significance, a two‐tailed *p* < 0.05 was selected.

## 3 RESULTS

### 3.1 Identifying DEFRGs and conducting enrichment analysis

**FIGURE 1:**
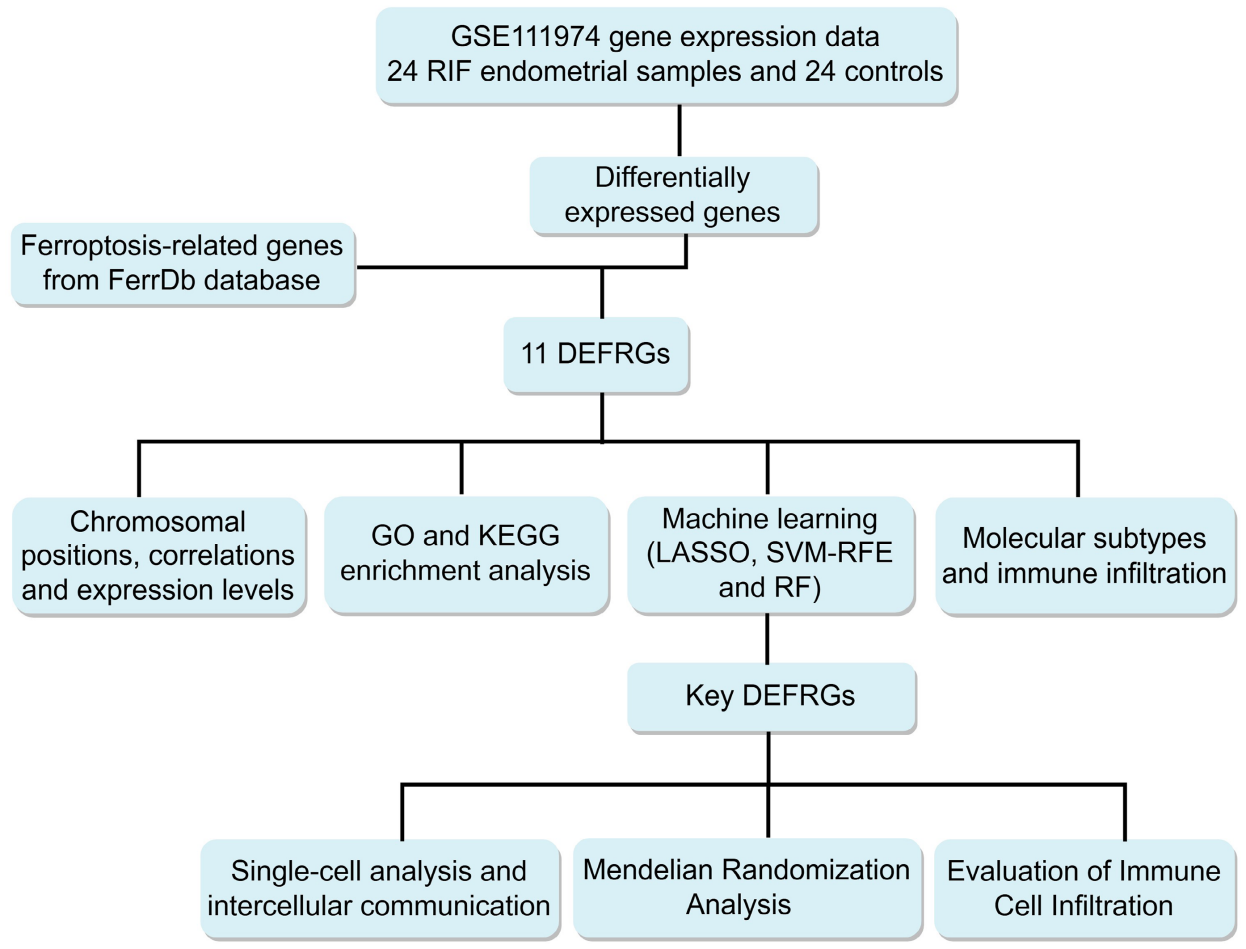
The flow chart for the study.

**FIGURE 2:**
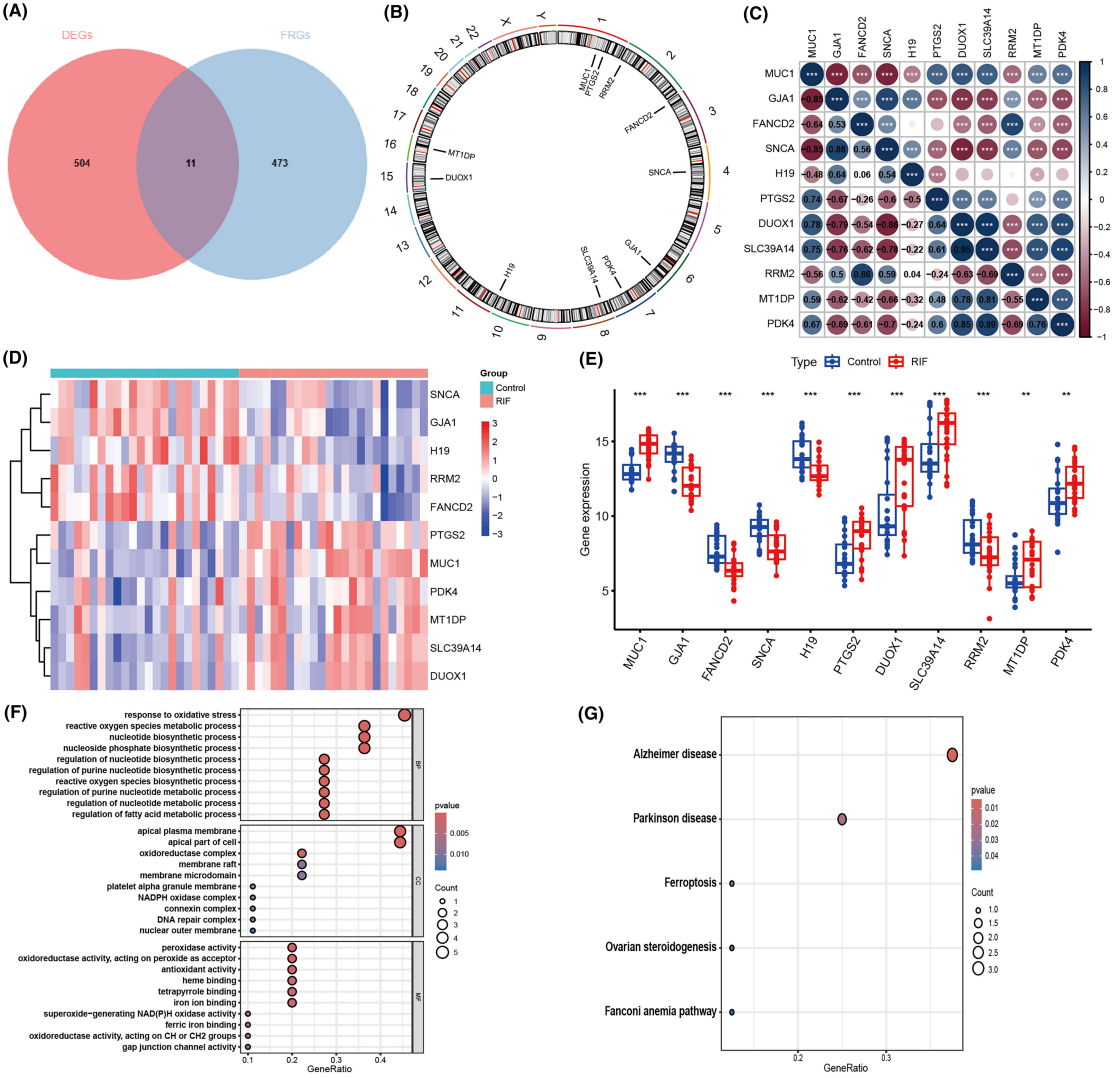
Landscape of 11 DEFRGs in RIF. (A) Identification of 11 DEFRGs. (B) The chromosomal positions of the 11 DEFRGs. (C) Spearman's correlation analysis of 11 DEFRGs. (D) Heat map of the expression of 11 DEFRGs between RIF patients and controls. (E) Histogram of the expression of 11 DEFRGs between RIF patients and controls. (F and G) GO and KEGG enrichment of 11 DEFRGs. *p < 0.05, **p < 0.01, ***p < 0.001.

Figure 1 showed the flow chart for the study. By intersecting 484 genes associated with ferroptosis and 515 DEGs, we identified 11 DEFRGs, which were represented in a Venn diagram (Figure 2A). Figure 2B showcased the chromosomal locations of the 11 DEFRGs in a circular diagram. In the end, the heat map and histogram were utilized to display variations in expression levels of the 11 DEFRGs. In RIF samples, we observed an upregulation of the expression levels of 6 DEFRGs and a downregulation of 5 DEFRGs compared to controls (Figure 2C,D). Detailed DEFRGs between RIF and controls were shown in Table S3. Figure 2E displayed a correlation heat map and the Spearman's correlation analysis exhibited that DUOX1 had the highest positive correlation with SNCA (cor = 0.86, *p* < 0.001), yet showed the most negative correlation with SLC39A14 (cor = −0.95, *p* < 0.001). The analysis of GO showed that the 11 DEFRGs were predominantly enriched in the gap junction channel activity, mitochondrial electron transport, and response to oxidative stress (Figure 2F). The analysis of KEGG revealed that the pathways enriched by 11 DEFRGs included Alzheimer's disease, Parkinson's disease, and Ferroptosis (Figure 2G).

### 3.2 Identification of molecular subtypes

**FIGURE 3:**
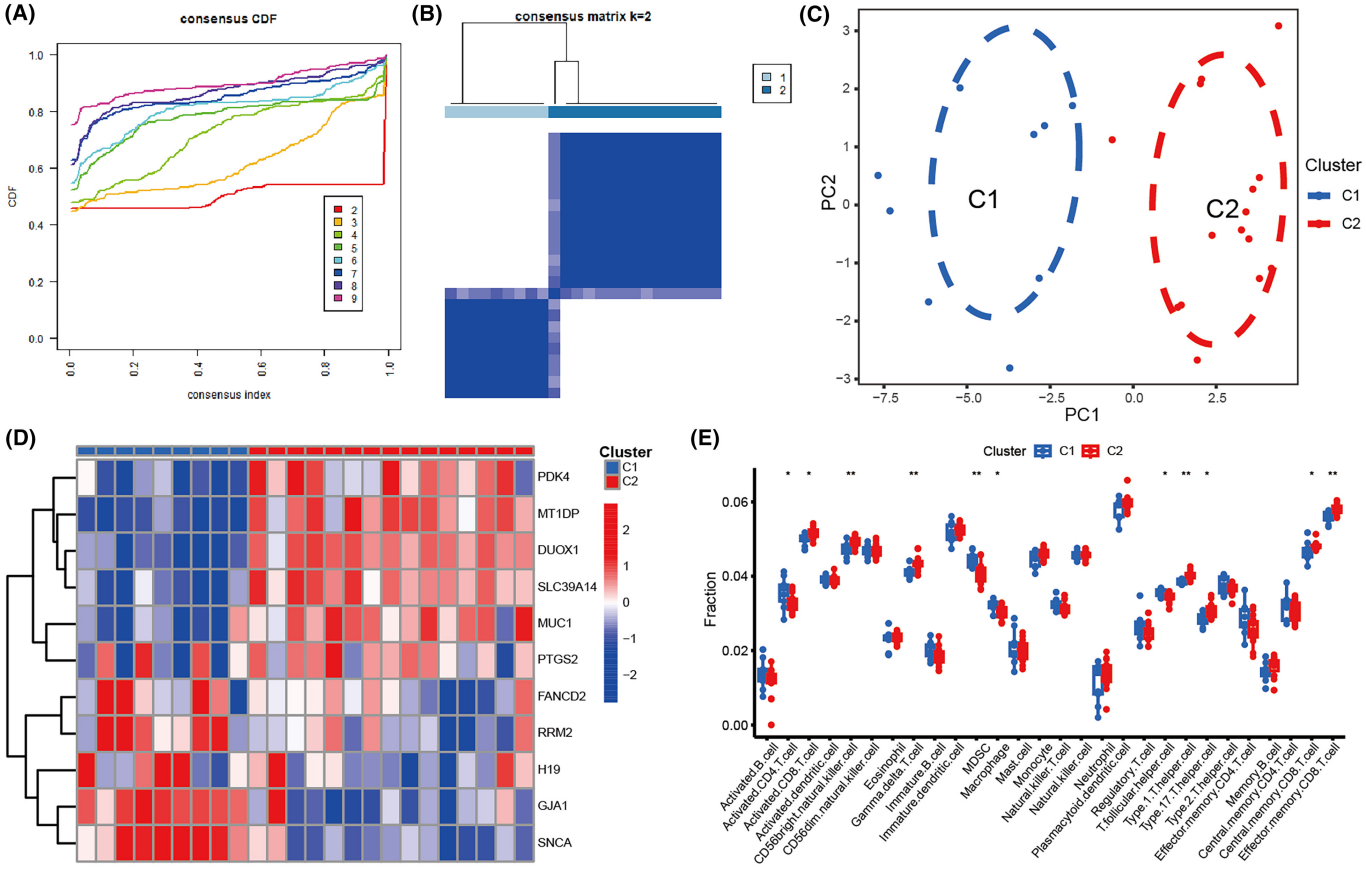
Molecular subtypes characterized based on 11 DEFRGs. (A) Heat map of 2 clusters (*k* = 2) derived from DEFRGs. (B) Cumulative distribution graph. (C) PCA analysis of the 2 clusters: Blue indicates cluster 1; red indicates cluster 2. (D) Heat map of gene expression levels between two clusters. (E) Different expression levels of immune cells between cluster 1 and cluster 2. *p < 0.05, **p < 0.01, ***p*P* < 0.001.

A total of 9 cluster results were determined based on 11 important biomarkers. Figure 3A–C were utilized as references to determine an appropriate molecular subtype based on the CDF value. The clustering outcome with the best results was when *K* = 2, and it was confirmed through PCA analysis. The correlation heat map indicated that GJA1, FANCD2, SNCA, H19 and RRM2 exhibited high expression levels in subtype 1, whereas MUC1, PTGS2, DUOX1, SLC39A14, MT1DP and PDK4 showed high expression levels in subtype 2 (Figure 3D). Detailed DEFRGs between C1 and C2 subtypes were shown in Table S4. In conclusion, we investigated the variances in immune microenvironment features between two subtypes (Figure 3E). The findings indicated that subtype 1 RIF exhibited elevated rates of immune cell infiltration, primarily in MDSCs, activated CD4 T cells, macrophages, and follicular helper T cells. Subtype 2 RIF exhibited increased infiltration abundance of CD56br natural killer cells, activated CD4 T cells, gamma delta T cells, type 1 helper T cells, effector memory CD8 T cells and central memory CD8 T cells.

### 3.3 Construction and evaluation of machine learning models

**FIGURE 4:**
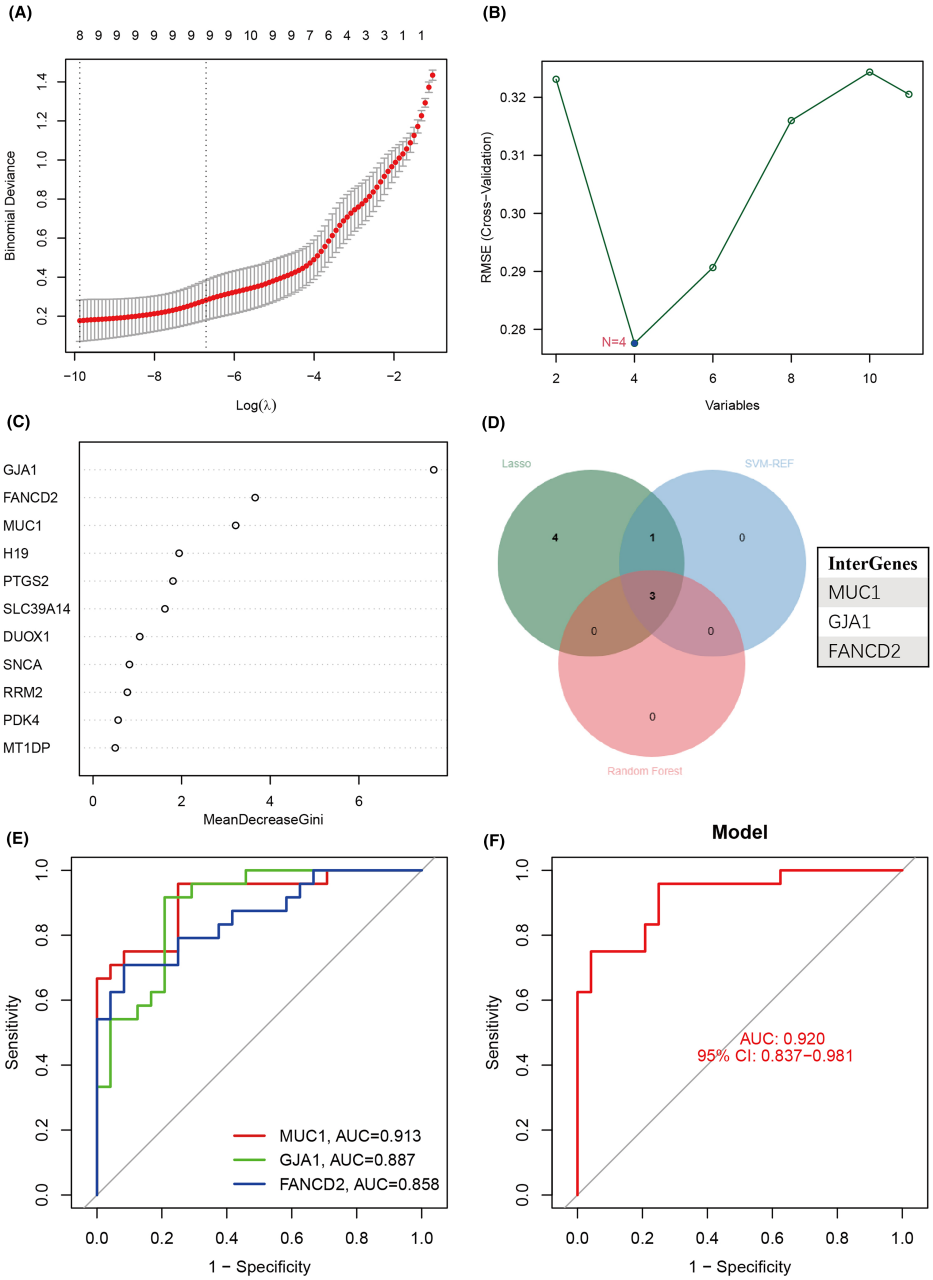
Detection of hub DEFRGs in RIF. (A) LASSO coefficient profiles of the eight co‐expressional DEFRGs. (B) The SVM‐RFE model selected 4 DEFRGs. (C) The RandomForest algorithm determined 3 DEFRGs with Gini>2. (D) Three hub DEFRGs were identified by machine learning methods. (E) ROC curves for the 3 diagnostic biomarkers. (F) A logistic regression model was performed to identify the RIF samples.

In order to determine credible diagnostic biomarkers associated with RIF, LASSO, SVM‐RFE and RF algorithms were utilized to assess 11 DEFRGs in RIF. Initially, by utilizing the least squares technique, we incorporated the gene expression patterns of 11 DEFRGs into LASSO regression. From the findings, it was discovered that eight possible DEFRGs were chosen, and the ideal lambda value was acquired (Figure 4A). Figure 4B showed that the SVM‐RFE method identified 4 DEFRGs that serve as reliable molecular biomarkers. Furthermore, the RF algorithm successfully identified 3 significant genes (Figure 4C). In RIF, three DEFRGs were identified, namely Mucin1 (MUC1), gap junction protein alpha 1 (GJA1) and Fanconi anaemia complementation group D2 (FANCD2). MUC1 was found to be upregulated, while GJA1 and FANCD2 were downregulated (Figure 4D). The AUC value for the intersecting DEFRGs exceeded 0.85, while the machine learning models achieved 0.92, demonstrating their accuracy in predicting RIF (Figure 4E,F).

### 3.4 Assessment of immune cell infiltration

**FIGURE 5:**
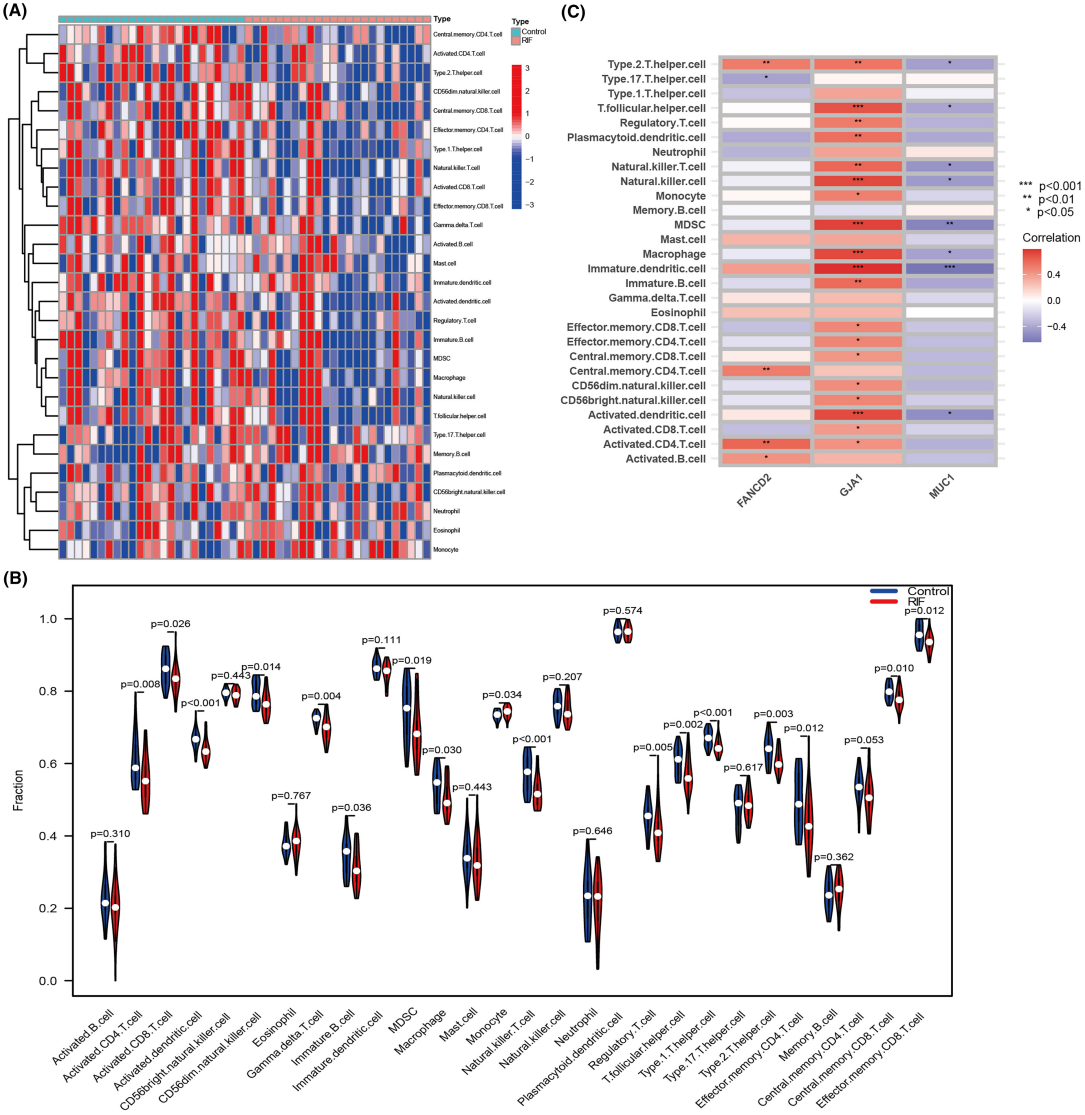
Evaluation of immune cell infiltration. (A) The ssGSEA heat map exhibited enriched immune cells in each tissue. (B) A violin graph showed the distinct fractions of immune cells in RIF group and controls. (C) Correlation between hub genes and immune cells. *p < 0.05, **P < 0.01, ***p < 0.001.

Immunological characteristics were estimated by the infiltration of immune cells. The heat map exhibited proportion of immune cells in each endometrium by ssGSEA score (Figure 5A). The violin graph revealed that the infiltration of immature B cells (*p* = 0.036), activated dendritic cells (DCs) (*p* < 0.001), regulatory T cells (Tregs) (*p* = 0.005), γδ T cells (*p* = 0.004) and myeloid‐derived suppressor cells (MDSC) (*p* = 0.019) was markedly lower in RIF patients than controls, while the monocytes (*p* = 0.034) was higher in RIF patients (Figure 5B). GJA1, an important molecule for cell gap junctions, is positively associated with almost all immune cells. FANCD2 was also positively related to the infiltration of type 2 T helper cells, activated CD4 T cells, activated B cells, and central memory CD4 T cell, while were negatively associated with type 17 T helper cells. Furthermore, MUC1 were negatively correlated with immature dendritic cells, MDSCs, helper T cells, natural killer cells macrophages and activated dendritic cells (Figure 5C).

### 3.5 Analysis of single‐cell RNA sequencing

**FIGURE 6:**
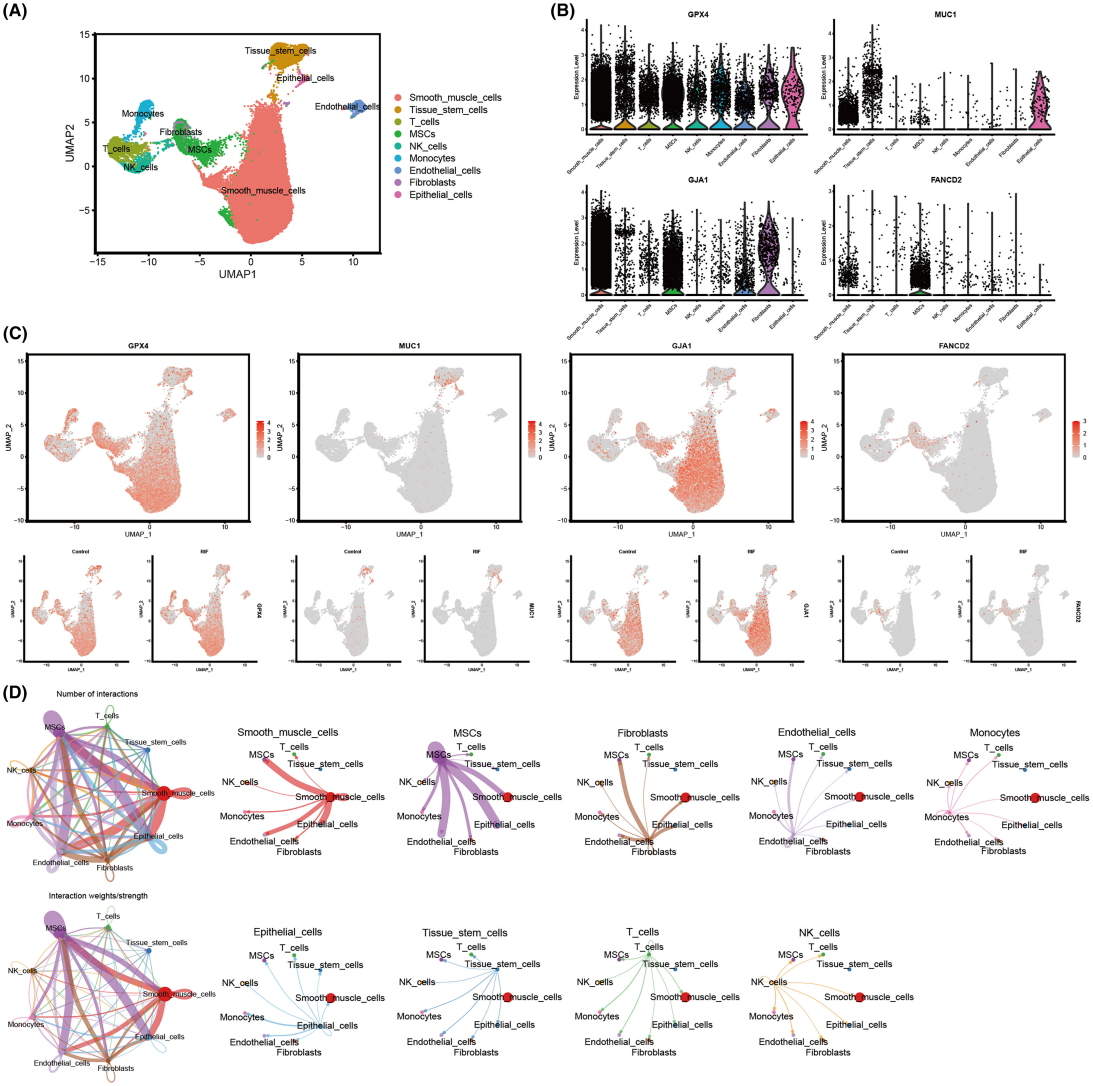
Diversity of cell types in the endometrium of RIF tissues and controls. (A) Nine cell clusters were identified on the UMAP plot. (B) The violin plots of three DEFRGs and GPX4 in various cells. (C) The UMAP plots of three DEFRGs and GPX4 in each cluster. (D) Among these hub genes, GPX4 (Log FC = 0.74), MUC1 (Log FC = 1.46), and GJA1 (Log FC = 0.61) are characteristic genes of endothelial cells. (E) The number and strength of interactions between 9 cell clusters.

In total, 67,378 cells were detected in the GSE183837 dataset after combining healthy endometrium (20,440 cells) and RIF endometrium (46,938 cells). Cells of poor quality were removed by applying a cut‐off of nFeature_RNA, 200, and a cut‐off of 40% for mitochondrial genes. Afterward, a total of 59996 cells, comprising healthy endometrium (18,403 cells) and RIF endometrium (41,593 cells), underwent screening for subsequent analysis. The dataset's available dimensions were identified through linear dimensionality reduction after choosing the top 2000 genes with high variability. The UMAP visualization was generated using the initial 15 principal components. In the end, we detected nine cell clusters, which were compared between two groups by generating UMAP plots (Figure 6A), consisting of Smooth muscle cells, Tissue stem cells, T cells, MSCs, NK cells, Monocytes, Endothelial cells, Fibroblasts and Epithelial cells (Table S6). Figure 6B,C display the violin plots and UMAP plots representing the expressed percentage of optimal DEFRGs in various cell clusters. Characteristics of Endothelial cells include GPX4 (log_2_FC = 0.74), MUC1 (log_2_FC = 1.46) and GJA1 (log_2_FC = 0.61) as optimal genes (Figure 6D). Significantly, GPX4 was detected in nearly every cell, whereas GJA1 predominantly resided in smooth muscle cells and fibroblasts, MUC1 primarily localized in epithelial cells and FANCD2 mainly resided in MSCs. Figure 6E displays the quantity and intensity of connections among 9 cellular clusters. It is important to mention that smooth muscle cells and MSCs played important roles in cellular communication in RIF cells. Furthermore, the MSCs exhibited a robust correlation with both epithelial and endothelial cells.

### 3.6 Mendelian randomization

**FIGURE 7:**
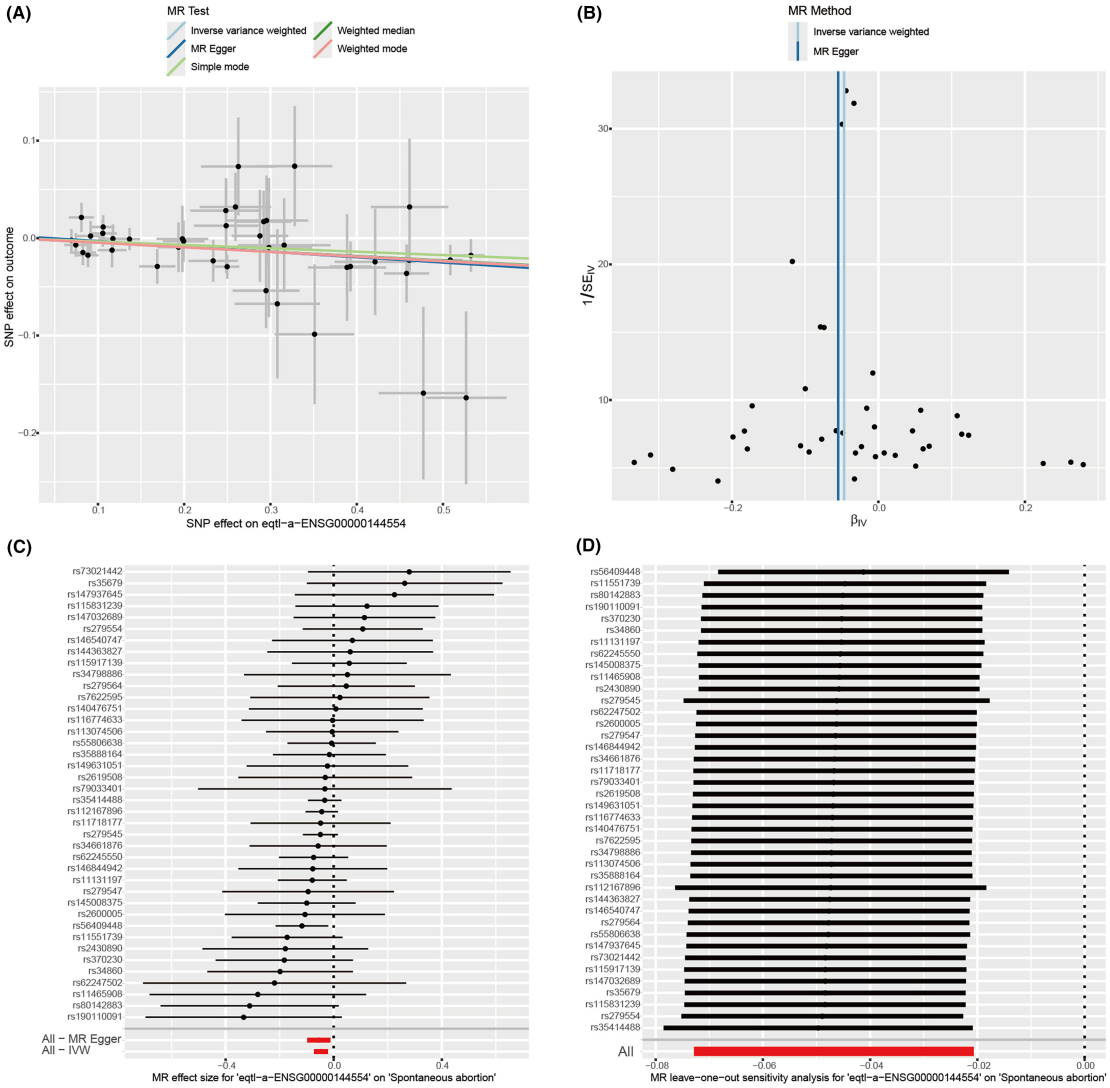
Mendelian randomization analysis of FANCD2 in RIF. (A) The scatter plot of the causal relationship between genes and disease using five different methods for analysis: Inverse variance weighted (IVW), MR‐Egger, weighted median, weighted mode, and simple mode. (B) The funnel plot displays the overall heterogeneity of the Mendelian randomization estimates for the effect of FANCD2 on RIF. (C) The forest plot of the causal relationship between each SNP and the risk of RIF. (D) The forest plot of the leave‐one‐out method.

The MR analysis investigated the causal relationship between four genes (GPX4, MUC1, GJA1, and FANCD2) and RIF. Results indicated that GPX4 and MUC1 did not exhibit a significant causal relationship with RIF (Table S5). Additionally, GWAS data for GJA1 were unavailable. Therefore, FANCD2 was chosen for detailed analysis. The population source for both FANCD2 and RIF GWAS data is European, ensuring consistency in ancestry. The identifier eqtl‐a‐ENSG00000144554 was retrieved using the ENSEMBL ID for the FANCD2 gene. A causal assessment showed the causal impacts of individual genetic variations on RIF (Figures 7A, 6C). The result of IVW method revealed that high levels of FANCD2 decreased the risk of RIF (*p* = 0. 001, OR = 0.954, 95% CI = 0.930–0.980). Notably, the causal effects demonstrated approximate symmetry in the funnel plot (Figure 7B), suggesting significant causal relationships across all analysed SNPs. Heterogeneity tests for IVW method yielded *Q*_*p* > 0.05, and the *p* value for horizontal pleiotropy test >0.05, indicating absence of heterogeneity and pleiotropy in Mendelian randomization. Additionally, the sensitivity analysis used by the leave‐out‐one method showed minimal fluctuations in the overall error bars (all error bars lie to the left of 0), suggesting that the results are robust (Figure 7D).

### 3.7 Validation in RIF patients

**FIGURE 8:**
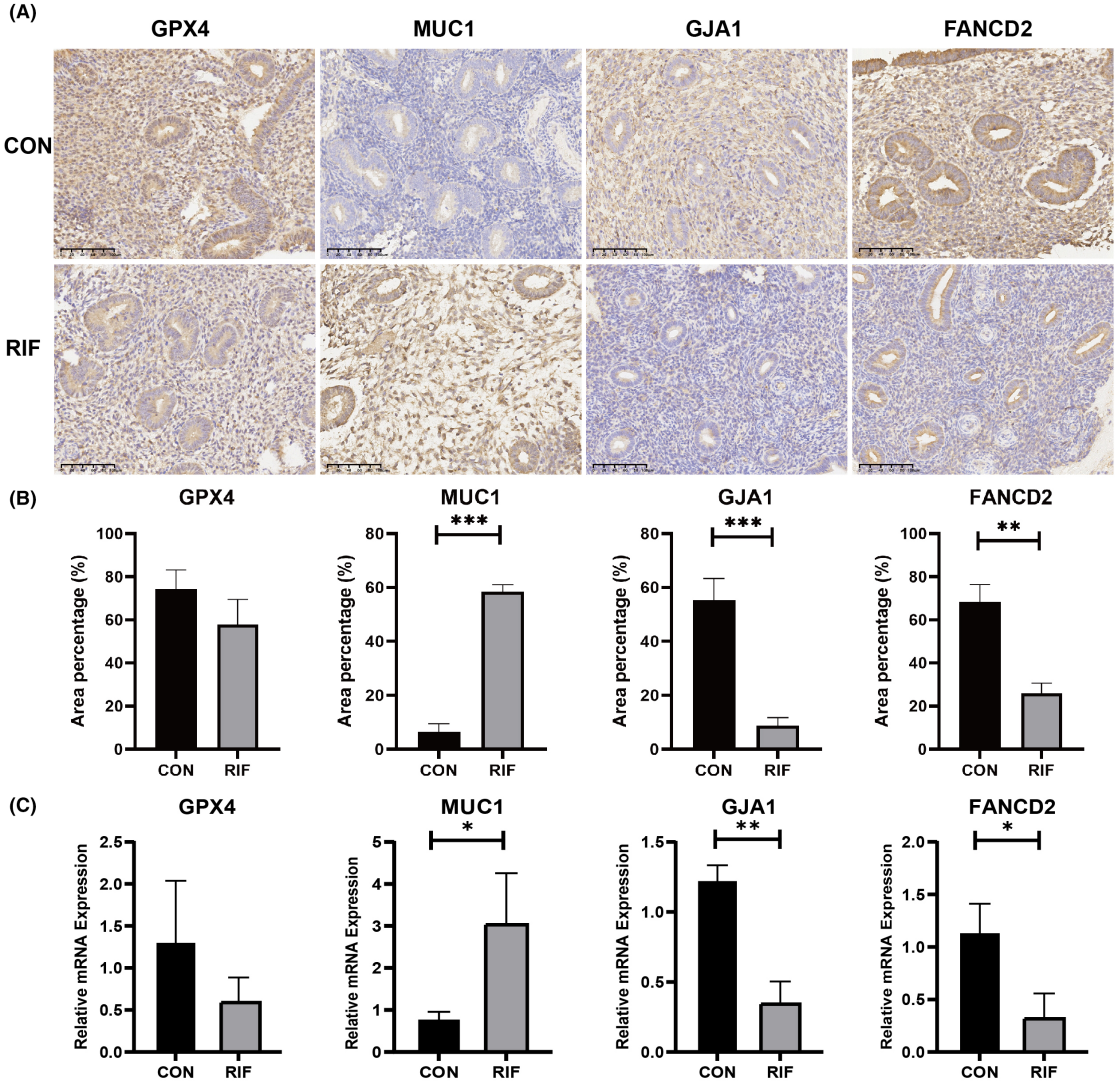
Validation of hub DEFRGs in clinical RIF endometrium. (A) 20× magnification regions of hub DEFRGs on immunohistochemically stained sections of RIF and control endometrial tissue. (B) Immunoreactive areas were quantitatively analysed by ImageJ. Five independent areas per slice, *n* = 5. (C) The endometrial expression of GPX4, MUC1, GJA1, and FANCD2 between RIF and control (CON) patients. *n* = 3, *P < 0.05, **P < 0.01, ***P < 0.001.

Using immunohistochemistry assay, we discovered the positive area percentage of four DEFRGs in endometrial tissues. We found MUC1 (*p* < 0.001) was significantly upregulated in RIF group, while GJA1 (*p* < 0.001) and FANCD2 (*p* < 0.01) were downregulated (Figure 8A,B). Meanwhile, we examined the mRNA expression levels of the four DEFRGs in endometrial tissues using qRT‐PCR. We found that MUC1 (*p* < 0.05) was significantly up‐regulated, while GJA1 (*p* < 0.01) and FANCD2 (*p* < 0.05) were downregulated in RIF group (Figure 8C). The results of Immunohistochemistry and qRT‐PCR were consistent with our bioinformatics analysis.

## 4 DISCUSSION

The process of embryo implantation plays a crucial role in human reproduction. The success of this step depends on blastocyst quality, endometrial tolerance, and successful crosstalk between the embryo‐maternal interface. RIF refers to the inability of embryos to successfully implant following multiple transfers. As the increased success rate of IVF, it has become increasingly difficult for patients to accept the outcome of transfer failure. Following the first report of ferroptosis in 2012,^28^ subsequent investigations have revealed a significant correlation between ferroptosis and conditions such as endometritis,^29^, ^30^ endometriosis,^31^, ^32^ recurrent miscarriage,^33^ and UI.^16^ However, as of now, no study has been reported on the relationship between iron overload, ferroptosis and RIF.

The present study first screened 11 DEFRGs, MUC1,^34^ GJA1,^35^ H19,^36^ PTGS2^37^ and RRM2^38^ have been demonstrated to have a notable impact on both embryo implantation and endometrial tolerance. MUC1, a glycoprotein with high expression in RIF, has been shown to be an inhibitor of ferroptosis.^39^ Zhang et al. found that erastin‐induced ferroptosis impedes endometriosis progression by targeting the MALAT1/miR‐145‐5p/MUC1 axis.^40^ We hypothesized that high MUC1 expression disrupts endometrial homeostasis by inhibiting endometrial cell ferroptosis, which in turn affects endometrial tolerance and leads to the development of RIF. However, there are also studies that contradict our conclusions. Bastu et al. showed that blood and tissue levels of MUC1 and glycosaminopeptide A were greatly reduced in women with RIF compared to women of childbearing age.^41^ Wu et al. reached the conclusion that endometrial MUC1 levels were notably decreased in patients with RIF in comparison to childbearing women during the implantation period.^42^ As a result, our hypothesis suggests that MUC1 might serve as an indicator to evaluate endometrial tolerance, and any abnormal MUC1 expression could result in compromised endometrial tolerance. Connexin 43 (Cx43), a product of the GJA1 gene, is a component of gap junction intercellular communication (GJIC), and is expressed predominantly in the endometrial stroma.^43^ Previous studies have shown that GJIC between endometrial stromal cells plays a critical role in regulating decidualization, neovascularization, and embryo implantation. Yu et al. have shown that disruption of GJIC can induce endometrial stromal cell apoptosis and lead to endometrial dysfunction.^44^ CX43, as a signal amplifier, is involved in death signal transduction in ferroptosis. Huang et al. found that inhibition of CX43 attenuates ferroptosis after spinal cord injury.^45^ Combined with our results, we speculate that the GJIC in the endometrium of patients with RIF is destroyed, ferroptosis signalling is blocked, and the endometrial microenvironment is destroyed, which in turn leads to the occurrence of RIF. H19 is a long non‐coding RNA that is normally expressed in the eutopic endometrium.^46^ Studies have shown that H19 expression is reduced in the eutopic endometrium of UI patients.^47^ Liu et al. reported that the expression of H19 in the endometrium of patients with RIF was significantly reduced in the mid‐luteal phase, indicating that H19 may have a potential role in regulating endometrial receptivity during the implantation window.^36^ Prostaglandin endoperoxide synthase 2 (PTGS2), involved in the conversion of arachidonic acid to prostaglandin H2, is expressed in inflammation.^48^ Wang et al. discovered that the endometrium of the RIF group exhibited a notable increase in the expression of PTGS2.^49^ Napolitani et al. demonstrated that overexpression of PTGS2 can directly act on memory CD4^+^ T cells, resulting in increased IL‐17 levels, which causes maternal immune rejection of the foetus and leads to implantation failure.^50^ Ribonucleoside‐diphosphate reductase subunit M2 (RRM2) is the rate‐limiting enzyme in the production of deoxynucleotides for DNA synthesis, which plays an important role in cell proliferation and tumorigenicity.^51^ Studies have shown that RRM2 may be an important effector of progesterone signalling to induce proliferation and decidualization of mouse uterine cells.^52^ FANCD2 acts as a suppressor of ferroptosis and holds significant significance in the advancement of cell cycle, replication, and repair processes.^53^ Earlier research has indicated that FANCD2 has a function in controlling the tumour microenvironment and impacting its spread, growth, and programmed cell death. Flaws in FANCD2 result in a rise in genetic material harm and the formation of cancer.^54^, ^55^ However, no investigations have established a connection between FANCD2 and the attachment of embryos. MUC1, GJA1 and FANCD2 were also recognized by machine learning algorithms. Thus, we speculate that dysregulated expression of three key DEFRGs leads to dysfunction of the ferroptosis signalling pathway, which in turn leads to abnormal execution of endometrial function.

Studies have demonstrated that an abnormal presence of immune cells in the endometrium can hinder embryo attachment and growth, resulting in infertility, miscarriage and other complications during pregnancy.^56^ And, ferroptosis is associated with dysregulated immune responses. Three DEFRGs were found to be associated with the infiltration of immune cells. Among them, GJA1 is positively correlated with almost all immune cells. CX43 can increase the production of Tregs^57^ and immunosuppressive potential.^58^ Our study reveals a direct association between GJA1 and Tregs. We also found that FANCD2 expression was positively correlated with the infiltration of activated CD4^+^ T cells and activated B cells. It has been reported that FANCD2 can affect the immune microenvironment of tumours. Huang et al. found that the presence of FANCD2 in hepatocellular carcinoma showed a strong correlation with an increased presence of CD4 T cells. And neutrophils, macrophages, CD8 T cells, DCs and B cells exhibited a similar pattern of infiltration, as reported in study.^59^ MUC1 was negatively correlated with the infiltration of MDSCs, helper T cells, natural killer cells, macrophages and activated DCs. Studies have shown that tumour‐related MUC1 glycosylation is closely related to DC recognition.^60^ MUC1 induces apoptosis in activated T cells, thereby inhibiting human T cell proliferation and cytotoxicity.^61^ MUC1 engages with neutrophils and macrophages in order to safeguard cancer cells throughout the process of metastasis.^62^ Extracellular vehicles (EVs) are important mediators of intercellular communication.^63^, ^64^ EVs can carry and deliver a variety of biologically active substances, including RNA, proteins, lipids and metabolites.^65^, ^66^ EVs secreted by endometrial cells of patients with RIF exhibit significant inhibitory effects in promoting embryonic growth and development.^67^ Studies have shown that EVs can transmit immune‐related signals through the systemic circulation.^68^ It has been confirmed that GJA1 is involved in the assembly of functional channels on the surface of EVs and the release of vesicle contents.^69^ MUC1 gene mutations can affect vesicle trafficking in renal epithelial cells.^70^ Therefore, we speculate that MUC1 and GJA1 may indirectly cause endometrial immune disorders by affecting the composition and release of extracellular vesicles. Subsequently, we conducted immunophenotyping of endometrial cells obtained from patients with RIF, which revealed that the expression of distinctive genes MUC1, GJA1 and FANCD2 in the C2 subtype aligned with the overall occurrence of RIF. Moreover, the C2 subtype had a higher immune activation, as shown by a higher level of infiltration of immune cell populations than the C1 subtype. Therefore, we concluded that the C2 subtype is an immune subtype and C1 is a non‐immune subtype in RIF. In summary, endometrial immune dysfunction plays an important role in the pathogenesis of RIF, and the three DEFRGs may be potential targets for RIF immunotherapy.

Finally, we utilized RIF single‐cell sequencing data, GWAS data and endometrial samples from RIF patients for validation. The result showed that the ferroptosis indicator (GPX4) was detected in almost all cells, and there was no significant difference in the genetic, mRNA, and protein levels between RIF and controls. During oxidative stress, GPX4 functions as glutathione peroxidase, dependent on both glutathione and selenium, to eliminate lipid hydroperoxides.^15^ There are currently two main surveillance mechanisms for inhibiting ferroptosis: one mediated by GPX4, which catalyses the reduction of phospholipid peroxides,^71^ and the other mediated by enzymes (such as FSP1) that generate antioxidant activity with free radical trapping Metabolites.^72^ Therefore, we speculate that ferroptosis of RIF endometrial cells may occur through a GPX4‐independent ferroptosis mechanism. Only FANCD2 showed a strong correlation to RIF in all analyses which may contribute to RIF pathogenesis through a non‐classical ferroptosis‐dependent pathway, providing new information for diagnosing and treating RIF in clinical practice.

Similarly, our study has some limitations. Initially, our findings came from bioinformatics examination. Further research, both in basic and clinical trials, is necessary to validate our discoveries. Second, due to the limitation of information in public databases, the sample size of the current study is insufficient and lacks a large validation dataset for overfitting check, which may lead to discrepancies between the results of the study and the actual situation. Additionally, our study lacks cellular functions of ferroptosis or ex vivo and in vivo animal experiments, so we will next begin to conduct relevant experimental studies.

## 5 CONCLUSION

This comprehensive study emphasize the significant role of DEFRGs in the pathogenesis of RIF, suggesting that modulating these genes could offer new avenues for treatment. The FANCD2 is a potential gene contributing to RIF pathogenesis through a non‐classical ferroptosis‐dependent pathway, providing a foundation for personalized therapeutic strategies in RIF management.

## AUTHOR CONTRIBUTIONS

**Yuanyuan Zhou:** Conceptualization (equal); formal analysis (equal); writing – original draft (equal). **Yujia Luo:** Formal analysis (equal); methodology (equal); writing – original draft (equal). **Wenshan Zeng:** Resources (equal). **Luna Mao:** Resources (equal). **Fang Le:** Data curation (equal). **Hangying Lou:** Methodology (equal). **Liya Wang:** Funding acquisition (equal). **Yuchan Mao:** Data curation (equal). **Zhou Jiang:** Writing – review and editing (equal). **Fan Jin:** Conceptualization (equal); writing – review and editing (equal).

## FUNDING INFORMATION

This research was funded by the National Key Research and Development Program of China, grant number 2018YFC1004900; the National Natural Science Foundation of China, Grant number 82001625.

## CONFLICT OF INTEREST STATEMENT

The authors declare no conflict of interest.

## Supporting information


Table S1.

## Data Availability

The GSE111974 and GSE183837 datasets supporting the conclusions of this article are available in the GEO database (http://www.ncbi.nlm.nih.gov/geo). The detail of GWAS database was presented in Table S1 (https://gwas.mrcieu.ac.uk/). The complete code for data analysis and data modelling on zenodo database (DOI: 10.5281/zenodo.13366618).
