# Supplementary material for: FANCD2 as a ferroptosis‐related target for recurrent implantation failure by integrated bioinformatics and Mendelian randomization analysis
Source: J Cell Mol Med. 2024 Oct 14;28(19):e70119. doi: 10.1111/jcmm.70119 (PMC11472029; doi:10.1111/jcmm.70119)
Supplement: Supplementary file 1 — Table S1. [file JCMM-28-e70119-s001.docx]

**FANCD2 as a ferroptosis-related target for recurrent implantation failure by integrated bioinformatics and mendelian randomisation analysis.**

**Supplementary materials**

**Table S1.** The source information of DEFRGs and Spontaneous abortion in GWAS.

**Table S2.** Primers applied to Quantitative Real-Time PCR.

**Table S3.** Identification of 11 DEFRGs between RIF patients and controls.

**Table S4.** Identification of 11 DEFRGs between cluster 1 and cluster 2 in RIF.

**Table S5.** Mendelian randomization estimates between genetically predicted DEFRGs and the risk of Spontaneous abortion.

**Table S6.** Markers for the nine cell type clusters, identified by the FindAllMarkers command in Seurat.

**Table S1.** The source information of DEFRGs and Spontaneous abortion in GWAS.

| **Categories of datasets** | **GWAS ID** | **Year** | **Sample size (case)** | **Number of SNPs** |
| --- | --- | --- | --- | --- |
| FANCD2 | eqtl-a-ENSG00000144554 | 2018 | 31,470 | 19,153 |
| MUC1 | eqtl-a-ENSG00000185499 | 2018 | 31,684 | 15,795 |
| GPX4 | eqtl-a-ENSG00000167468 | 2018 | 31,470 | 20,876 |
| Spontaneous abortion | O15_ABORT_SPONTAN | 2023 | 181667 (18680) | 19,672,091 |

**Table S2.** Primers applied to Quantitative Real-Time PCR.

| Primers | Sequence (5′-> 3′) | |
| --- | --- | --- |
| GPX4 | Forward | 5′- GAGGCAAGACCGAAGTAAACTAC -3′ |
|  | Reverse | 5′- CCGAACTGGTTACACGGGAA -3′ |
| MUC1 | Forward | 5′- TGCCGCCGAAAGAACTACG -3′ |
|  | Reverse | 5′- TGGGGTACTCGCTCATAGGAT -3′ |
| GJA1 | Forward | 5′- CAATCTCTCATGTGCGCTTCT -3′ |
|  | Reverse | 5′- GGCAACCTTGAGTTCTTCCTCT -3′ |
| FANCD2 | Forward | 5′- AAAACGGGAGAGAGTCAGAATCA -3′ |
|  | Reverse | 5′- ACGCTCACAAGACAAAAGGCA -3′ |
| GAPDH | Forward | 5′-GCCTCAAAATCCTCTCGTTGTG-3′ |
|  | Reverse | 5′-GGAAGATGGTGATGGGATTTC-3′ |

**Table S3.** Identification of 11 DEFRGs between RIF patients and controls.

| **id** | **logFC** | **AveExpr** | **t** | **P.Value** | **adj.P.Val** | **B** |
| --- | --- | --- | --- | --- | --- | --- |
| MUC1 | 1.686303 | 13.85347 | 7.088489 | 4.36E-09 | 7.85E-07 | 10.67968 |
| GJA1 | -1.70463 | 13.10748 | -6.03726 | 1.91E-07 | 1.51E-05 | 7.032057 |
| FANCD2 | -1.30901 | 7.016256 | -4.93374 | 9.34E-06 | 0.000292 | 3.298045 |
| SNCA | -1.18892 | 8.51589 | -4.48369 | 4.30E-05 | 0.000945 | 1.840972 |
| H19 | -1.15217 | 13.56438 | -3.93799 | 0.000255 | 0.003657 | 0.155245 |
| PTGS2 | 1.401311 | 7.933471 | 3.835996 | 0.000352 | 0.004634 | -0.14782 |
| DUOX1 | 2.191979 | 11.47839 | 3.194731 | 0.002424 | 0.019153 | -1.94674 |
| SLC39A14 | 1.560333 | 14.93633 | 3.141574 | 0.002822 | 0.021456 | -2.08661 |
| RRM2 | -1.14098 | 8.028238 | -2.76453 | 0.007961 | 0.045272 | -3.03213 |
| MT1DP | 1.006101 | 6.281409 | 2.676198 | 0.010041 | 0.053098 | -3.24108 |
| PDK4 | 1.077217 | 11.70793 | 2.642626 | 0.010954 | 0.056313 | -3.31917 |

**Table S4.** Identification of 11 DEFRGs between cluster 1 and cluster 2 in RIF.

| **id** | **logFC** | **AveExpr** | **t** | **P.Value** | **adj.P.Val** | **B** |
| --- | --- | --- | --- | --- | --- | --- |
| DUOX1 | 4.495522 | 12.57438 | 10.74278 | 3.49E-12 | 3.84E-11 | 17.80698 |
| MT1DP | 2.772703 | 6.784459 | 8.692911 | 5.84E-10 | 3.21E-09 | 12.73802 |
| SLC39A14 | 2.946933 | 15.7165 | 7.617561 | 1.06E-08 | 3.90E-08 | 9.856885 |
| SNCA | -1.67504 | 7.921429 | -5.97926 | 1.12E-06 | 3.08E-06 | 5.236315 |
| GJA1 | -1.65182 | 12.25517 | -5.26004 | 9.14E-06 | 2.01E-05 | 3.165221 |
| PDK4 | 1.925844 | 12.24654 | 5.168314 | 1.19E-05 | 2.19E-05 | 2.901567 |
| MUC1 | 1.301305 | 14.69662 | 4.444313 | 9.78E-05 | 0.000154 | 0.841739 |
| RRM2 | -1.64652 | 7.45775 | -3.16968 | 0.033365 | 0.045876 | -2.55295 |
| PTGS2 | 0.928946 | 8.634126 | 1.967322 | 0.057802 | 0.070647 | -5.15226 |
| FANCD2 | -0.65096 | 6.36175 | -1.80208 | 0.080893 | 0.088982 | -5.43893 |
| H19 | -0.56433 | 12.98829 | -1.5163 | 0.13919 | 0.13919 | -5.88527 |

**Table S5.** Mendelian randomization estimates between genetically predicted DEFRGs and the risk of Spontaneous abortion.

| **Exposure**  **(Gene)** | **Outcome** | **SNPs** | **Methods** | **OR (95%CI)** | **P**  **value** | **Egger**  **intercept**  **(p_value)** | **Cochran’s Q test-IVW** | | |
| --- | --- | --- | --- | --- | --- | --- | --- | --- | --- |
|  |  |  |  |  |  |  | **Q** | **Q_df** | **Q_P**  **value** |
| FANCD2 | Spontaneous  abortion | 40 | IVW | 0.954 (0.930-0.980) | 0.001 | 0.643 | 30.685 | 39 | 0.827 |
|  |  |  | WM | 0.955 (0.919-0.992) | 0.018 |  |  |  |  |
|  |  |  | MR-Egger | 0.947 (0.906-0.988) | 0.017 |  |  |  |  |
| MUC1 | Spontaneous  abortion | 34 | IVW | 0.977 (0.938-1.017) | 0.255 | 0.386 | 26.319 | 33 | 0.789 |
|  |  |  | WM | 0.985 (0.931-1.041) | 0.584 |  |  |  |  |
|  |  |  | MR-Egger | 1.002 (0.934-1.074) | 0.961 |  |  |  |  |
| GPX4 | Spontaneous  abortion | 51 | IVW | 1.015 (0.994-1.036) | 0.156 | 0.171 | 61.843 | 50 | 0.122 |
|  |  |  | WM | 1.039 (1.011-1.068) | 0.006 |  |  |  |  |
|  |  |  | MR-Egger | 1.038 (0.999-1.078) | 0.058 |  |  |  |  |

**Table S6.** Markers for the nine cell type clusters, identified by the FindAllMarkers command in Seurat.

| Smooth muscle cells | Tissue stem cells | T cells | MSCs | NK cells | Monocytes | Endothelial cells | Fibroblasts | Epithelial cells |
| --- | --- | --- | --- | --- | --- | --- | --- | --- |
| PDGFRB | CD49F | CD3D | CD73 | PTPRC | PTPRC | CLDN5 | HOXA10 | KRT18 |
| MCAM | CD71 | CD3E | CD90 | NACM1 | CD14 | PECAM1 | MME | KRT8 |
| SUSD2 | KRT15 | CCR7 | CD105 | NKG7 | TYROBP | VWF | DCN | EPCAM |
